# Both technological innovations and cultural change are key to a sustainability transition

**DOI:** 10.1371/journal.pbio.3002298

**Published:** 2023-09-21

**Authors:** Navin Ramankutty

**Affiliations:** Institute for Resources, Environment and Sustainability & School of Public Policy and Global Affairs, University of British Columbia, Vancouver, Canada

## Abstract

Can technology will solve our sustainability problems, or do we just need to reduce our consumption? In this Perspective, Navin Ramankutty argues that the answer is both, not just because it will be the most effective, but because they are needed for different timeframes and reasons.

## Teaser

Transitioning to sustainability will require technological innovations in the short term, but also cultural change to embrace traditional and Indigenous ideas of respect, responsibility, sufficiency, and reciprocity to reduce consumption in the long term.

We are said to be living in the Anthropocene, a time when human activities are having as great an impact on the Earth system as other geological forces. According to the “Planetary Boundaries” framework, which uses the past 10,000 years (the Holocene) as a benchmark, human influence on the Earth system has greatly exceeded the “safe operating space” across multiple indicators, including climate change, biodiversity loss, and nutrient pollution [1]. A critical message is that even if we solve the climate problem, the biodiversity and nutrient pollution challenges will remain.

Taking biodiversity loss, its biggest cause is habitat loss for plants and animals because of land-use change [2]. And the biggest cause of land-use change is agriculture [3]. So the main leverage for addressing the biodiversity crisis is through modifying land use for agriculture.

How do we do that? We essentially have 2 options. One is to limit the amount of land used for farming by intensifying agriculture. Technology has greatly increased the productivity of agriculture since the 1940s by increasing inputs (e.g., through use of irrigation and fertilizers) and through the development of new seed varieties [3]. Corn yields in the United States of America, for example, increased 6-fold, from approximately 2 tonnes/ha during 1866 to 1940 to nearly 12 tonnes/ha in 2022. Similarly, the Green Revolution increased wheat and rice yields in Asia and Latin America since the 1960s [3]. But input intensification itself can exacerbate biodiversity loss and also lead to the depletion of freshwater, soil degradation, nutrient pollution, and greenhouse gas emissions [3].

Alternatively, we could adopt agroecological farming practices that tread more lightly on the land [4]. Agroecological practices (e.g., intercropping and agroforestry) aim to incorporate ecological processes such as natural pest regulation and biological nitrogen fixation but are often dismissed for having lower yields. Organic agriculture (used here as a proxy for agroecology in the absence of broadscale assessments of the latter) has many environmental benefits per unit area; however, the environmental benefits per unit product are comparable to conventional farming due to its approximately 20% lower yields [5]. In other words, for growing the same amount of food, organic farms are not discernibly better for the environment than conventional farms (although more research and development investment in organic farming might close yield gaps, and organic also has other benefits such as limiting pesticide exposure).

Debate has raged over these 2 alternative strategies and their trade-offs, popularly labeled “land sparing versus land sharing” [3]. But the debate on which alternative is better has missed an important consideration of just how much better. Ultimately, my opinion is that we are fiddling at the margins with either approach when thinking about food system sustainability from a purely supply perspective [6]. The sheer magnitude of the number of people on this planet, and more importantly, our consumption, is driving environment impacts, and small improvements at the margins of production systems will not make a big difference. Such “fiddling at the margins” of production systems is common across many domains, and is maybe why, despite expanding renewable energy and setting aside protected areas, the planet keeps warming and biodiversity keeps crashing. Where does that leave us?

Many argue for transformative change [7]. What does this mean? For some, it means transforming our production practices and economic systems to be more environmentally friendly. For me, it means the need to take a much harder look at the very basis of our consumption. Ten billion people consuming as we do just cannot engineer or model our way out of this crisis. We need to not only change how we produce our food, but to also reduce our consumption. Today’s crop harvests deliver only approximately 60% of their calories to humans, the rest is lost to the metabolism of livestock and to bioenergy [8]. Shifting to plant-based diets can be good for the planet and also good for human health [9]. We also need to reduce food waste, given that approximately 30% is wasted. Transformative change is also important because our consumption touches on multiple environmental crises at the same time. Without transformative change, we might end up solving one problem while making another worse, or creating new ones. For example, nuclear fusion may help to solve the climate problem but the plentiful availability of carbon-free energy will enhance our capacity to clear and modify the land even more and may exacerbate the biodiversity and nutrient pollution problems. Finally, transformative change is critical to addressing the massive inequalities in society (economic, environmental, and food-related, among others).

As we think about what transformative change means, we may do well to embrace some principles from the peoples who have had a sustained and long history of responsible resource use—Indigenous Peoples and traditional local communities. These traditional communities have had a very different relationship with the land and resources than modern societies do. Their knowledge–practice–belief system, built on centuries of “diachronic observations” [10], emphasizes the coevolution of humans and nature and embodies respect for the environment and responsible use of land and resources over multiple generations, including never taking more from the land than needed and giving back to the land in reciprocity for what you take from it [11]. So transformative change is about changing cultural norms to embrace important ideas of respect, responsibility, sufficiency, and reciprocity.

Whenever the topic of traditional and Indigenous Knowledge comes up, there is the common refrain, “but we cannot go back”. Human civilizations have essentially gorged on natural resources and grown our numbers, and we are approaching 10 billion people. We have also improved our wellbeing and achieved a high quality of life that we do not wish to give up (although inequality has worsened and extreme excess is widespread in some parts of the world). There is path dependency to our problems. So we can only go forwards. Is the Holocene (the basis of the Planetary Boundaries framework) a good benchmark for the Anthropocene? How relevant are lessons of the past in going forward? What new lessons do we need to learn?

**Fig 1:**
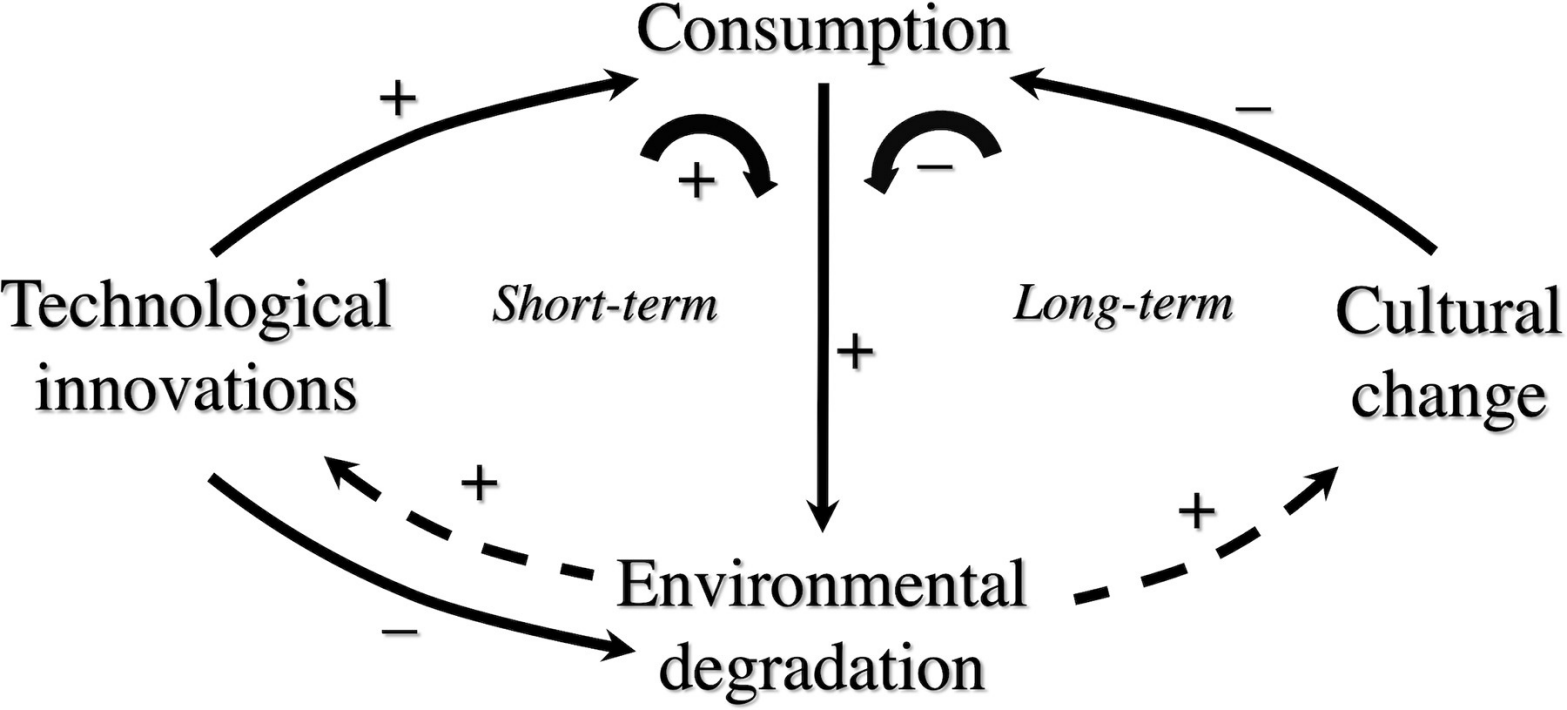
Interactions between technology, culture, consumption, and environment. The solid arrows show the influence of technological innovations and culture on consumption, of technology on environment, and of consumption on environment, while the dashed arrows show the societal responses (i.e., increased use of technology or cultural shifts) to environmental degradation. Technological innovations (technologies explicitly designed to minimize environmental harms and that are realistically available and not a chimera (e.g., nuclear fusion) promised in the future) are urgently needed to address our environmental challenges. However, such technological innovations, in the long term, can also increase consumption and increase environmental harm. We need cultural change (principles such as respect, responsibility, sufficiency, and reciprocity) to reduce our consumption in the long term.

I do not have answers to these questions, but here is what I believe (Fig 1). First, in the short term, we have numerous sustainability challenges that need to be addressed urgently. Failing to do so will result in massive human suffering, especially by those who are most disadvantaged. We cannot forgo the opportunity provided by technological innovations [12], for example, genetic engineering or plant-based meat alternatives, to reduce our environmental impacts in the short term. Increasing the efficiency of agricultural productivity growth (i.e., more output per input) can improve the sustainability and resilience of agriculture [13]. But technological innovations can also boost overall consumption (Jevons paradox); to avoid that, in the long term, we will need shifts in cultural norms, from a growth mindset to one of sufficiency, and for wellbeing to be more about quality of time than about abundance of goods.

Many conversations today pit technology against social change, often with good reason. There are techno-utopians who believe technology can fix all our sustainability challenges and see no need to limit consumption, and conversely techno-dystopians who do not see any role for innovative new technologies. Neither of these extreme views is going to address our sustainability transition challenges. Instead, we need to simultaneously embrace key technological innovations in the short term to minimize human suffering and environmental harm and prevent overshoot of Earth’s carrying capacity and long-standing and land-based knowledge and wisdom in the long term to shift norms and behaviors to reduce our consumption (Fig 1).
